# Assessment of tissue glycation on plantar soft tissue stiffness

**DOI:** 10.1186/1757-1146-7-S1-A83

**Published:** 2014-04-08

**Authors:** Jee Chin Teoh, Taeyong Lee

**Affiliations:** 1 Department of Biomedical Engineering, National University of Singapore, Singapore

## Introduction

Tissue glycation, that occurs naturally through ageing and can be sometimes accelerated by disease such as diabetes mellitus, is clinically claimed to have induced irregular collagen alignment and increased collagen fibril density in patients [1]. This hence increases tissue stiffness and leads to plantar injury, i.e. ulcer. In the USA, 85% of all non-traumatic amputations in diabetes patients arise from non-healing ulcers [2]. This tells the need to assess and to detect tissue abnormality early, in order to prevent problematic tissue rupture especially in elderly and diabetes subjects. Currently, there are several existing tools used by clinicians like monofilament, tuning forks, biothesiometers, neurothesiometers etc. However, majority of them only measure subjective sensing ability but not the mechanical property of the plantar tissue. The objective of this study is to investigate the effects of (i) natural tissue glycation (ageing) and (ii) accelerated tissue glycation (diabetes mellitus) on plantar soft tissue stiffness using the proposed indenter [3].

## Methods

First experiment investigates the plantar tissue stiffness as a consequence of natural ageing. 25 young (22±1.6 yrs) and 25 old subjects (67±5.8 yrs) of similar physical attributes are recruited. Second experiment involves 35 normal and 5 diabetic subjects of similar physical attributes and ages. It assesses the effect of accelerated tissue glycation due to diabetes on plantar tissue stiffening. During stiffness measurement, indentor tip probes the plantar soft tissue to obtain localized force response underneath the 2nd metatarsal head pad at 3 different dorsiflexion angles of 0°, 20°, 40° and the hallux and heel at 0°. Maximum tissue deformation is fixed at 5.6mm (close to literature data) [4].

## Results

**Figure 1:**
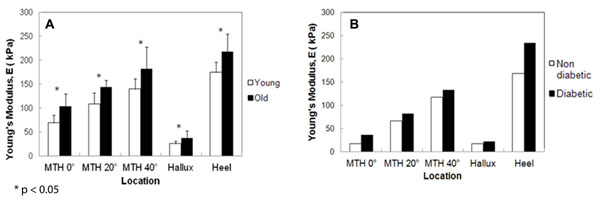
Comparison of plantar STS between (A) young and elderly; (B) diabetic and non diabetic subjects. *p < 0.05

Tissue responses are compared (Fig. 1 A&B). Old subjects show significantly higher tissue stiffness in all foot sites tested with p<0.05. Diabetes subjects are found to have stiffer plantar tissue in foot regions tested.

Both natural and accelerated tissue glycation stiffen plantar soft tissue resulting in stiffer and weaker tissue property. This study successfully demonstrates the ability of proposed indentation technique to quantify positive relationship between tissue glycation and plantar soft tissue stiffness.
